# Cognitive and autonomic dysfunction measures in normal controls, white coat and borderline hypertension

**DOI:** 10.1186/1471-2261-11-3

**Published:** 2011-01-11

**Authors:** Abdullah Shehab, Abdishakur Abdulle

**Affiliations:** 1 Division of Medical Sciences, Queen Elizabeth Hospital, University Hospitals Birmingham NHS Trust, (Queen Elizabeth), Birmingham, (B15 2TH), UK; 2 Department of Internal Medicine, Faculty of Medicine and Health Sciences, UAE University, (Maqam), Al-Ain, (P. O. Box: 17666), United Arab Emirates

## Abstract

**Background:**

White coat hypertension (WCHT) is a significant clinical condition with haemodynamic differences and presence of functional changes. We aim to compare cognitive and autonomic dysfunction variables (heart rate variability) between subjects with normal blood pressure (controls), WCHT, and borderline hypertension (BLH).

**Methods:**

We performed a cross-sectional study in a cohort of 69 subjects (mean age ± SD; 38.2 ±10.8 years) comprising comparable number of normal controls, WCHT, and BLH. We measured clinic and 24-hour ambulatory blood pressure monitoring (ABPM), cognitive function parameters, and heart rate variability (HRV). All subjects underwent 24-hour ambulatory electrocardiography monitoring which was analyzed for HRV measurements. We performed a routine echocardiography (ECHO) for all subjects.

**Results:**

Multiple comparison between the three groups revealed significant (p < 0.04) differences in mean day-time ABPM (systolic and diastolic). In the state anxiety inventory (SAI), both subjects with WCHT and BLH had significantly (p < 0.006) higher anxiety levels than the control group. In memory tasks WCHT subjects scored significantly (p < 0.004) lower in comparison with the other two groups. WCHT significantly (p < 0.001) performed less in memory tests, whereas BLH subjects had significantly (p < 0.001) lower reaction time. We found a significant (p < 0.05) difference in the 24-hour RMSSD and SDNN between the three groups. There was significant correlation between 24-hour RMSSD and computer CANTAB scores. The Echocardiography assessment revealed no significant differences in LV mass indices and diastolic function.

**Conclusions:**

WCHT and BLH subjects showed lower cognitive performance and higher levels of anxiety when compared to controls. Autonomic function reflected by HRV indices was lower in WCHT and BLH in contrast to control, though not significantly. Our results suggest that WCHT may not be a benign condition as it may contribute to the overall risk for cardiovascular disease and LV damage. Longitudinal studies of patients with WCHT should clarify the transient, persistent or the progressive nature of this condition.

## Background

White coat hypertension (WCHT), a transient increase in blood pressure (BP) measured in a medical setting, is a significant clinical condition with haemodynamic differences and presence of functional vascular changes and increased peripheral resistance [1,2]. It is not clear whether WCHT is a variant of hypertension [3] or a rise in BP during stress [4] especially with regard to cognitive and autonomic dysfunction variables.

To overcome BP fluctuations, ambulatory blood pressure monitoring (ABPM) over a 24-hour period may provide more accurate BP readings [5]. However, the prognostic significance of ABPM, indicates that there is no increased cardiovascular risk among WCHT [6].

Epidemiological studies showed that drug treatment lowered clinic pressure in all patients but had little effect on the ABPM among patients with WCHT [7,8]. Hence, the assumption that office hypertension should not be treated in patients with "normal" ABPM may need to be revisited. In part, because high office BP is the basis of the existing evidences that hypertension is harmful, and that treatment of hypertension is indeed beneficial [9]. Although WCHT is of advantage compared to masked and sustained hypertension, it is nevertheless up-normal [10]. We hypothesize that assessing the cognitive function and heart rate variability (HRV) may be of importance in identifying subjects at higher risk of developing hypertension.

Existing evidence shows that patients with hypertension performed poorly than normotensive subjects across multiple domains of neuropsychological function including memory and attention tasks, awareness of bodily sensations, and cognitive function; reasoning and memory [11,12]. More recent research has highlighted that greater variability in BP on 24-hour ABPM serves as an index of autonomic deregulation and is associated with poorer cognitive performance [13].

HRV, known to be associated with development of many cardiac and non-cardiac diseases [14,15], has been implicated as a predictor of hypertension [16]. On the contrary, no differences were shown in HRV among WCHT as compared to both patients with hypertension and control subjects [17]. Regardless, population-based studies indicate increased sympathetic activity among WCHT in comparison to masked and sustained hypertension [18].

The magnitude of BP increase during mental stress predicts progression of atherosclerosis more strongly than baseline BP, cholesterol level, and history of smoking [19]. Even so, while some studies have shown that target organ damage is less prevalent [20], other studies reported higher-than-normal left ventricular mass (LVM) among WCHT patients [21]. As a result, WCHT may be considered as an intermediate condition between normotension and hypertension in relation to target organ damage including carotid stiffness and left ventricular hypertrophy [22].

Therefore, this study aims to compare important clinical indicators i.e. clinic BP, ABPM, cognitive function, and HRV, across three groups; subjects with normal BP, WCHT, and BLH.

## Methods

### Study design

In this cross-sectional study, we recruited a total of sixty nine individuals including control subjects (n = 20), WCHT (n = 19), and BLH (n = 30) from among healthy normal volunteers and visitors of the outpatient hypertension clinic at Wellcome Trust clinical research facility, University Hospital Birmingham NHS Trust, Birmingham, United Kingdom.

### Subjects

Healthy controls were defined as subjects with normal BP < 130/80 mm Hg in the clinic and during the day-time and in the absence of other risk factors. WCHT was defined as patients with clinic BP > 140/90 mm Hg, but with ambulatory day-time BP < 135/85 mm Hg. BLH was defined as patients with systolic BP > 140 mm Hg and < 160 mm Hg and/or diastolic BP > 90 mm Hg and < 99 mm Hg both in the clinic and during the day-time. The Research and Ethics Committee of the University of Birmingham approved the study protocols prior to subject recruitment. All subjects gave written and informed consent to participate in the study.

### Methods

Demographic and anthropometric variables as well as history of smoking, alcohol consumption, and family history of heart disease (HD) were recorded. All subjects referred to the clinic had 24-hour ABPM (Spacelabs Medical, model 90217-validated).

Clinic BP, was measured in the sitting position by standard oscillometeric sphygmomanometer (Dinamp, Critikon) with an appropriate cuff size (Dura-cuff, Johnson & Johnson Ltd) attached to the participant's upper left arm. The average day-time pressure was used to determine BP status. Account was taken for the differences between ABPM measurements and clinic measurements by the addition of 12/7 mm Hg to the mean day-time pressure taken by ABPM according to guidelines [23]. BP levels were classified according to the British Hypertension Guidelines [24].

Cambridge Neuropsychological Test Automated Battery (CANTAB) computer tests were evaluated in the three groups by a battery of 5 computerized tests including paired associates learning task (PALT), spatial recognition and memory task (SRMT), reaction time (RT 11), spatial span test (SSPT), and spatial working memory (SWMT) together with an additional 3 paper format tests namely Alice Heim 4 (AH4) verbal and numerical reasoning test, paced auditory serial addition test (PASAT) and state anxiety inventory (SAI) test - according to standard procedures.

As detailed previously [25], we measured HRV from 24-hour electrocardiography monitoring calculated in the time domain and according to standard procedures [26]. Time-domain measures included the overall variability in the entire recording, calculated as the standard deviation (SD) of all normal R-R intervals (SDNN) and the mean square root of successive differences (RMSSD). The triangular index was obtained by dividing the total number of all R-R intervals by the height of the histogram of all R-R intervals, measured on a discrete scale with bins of 7.8 ms. Long-term HRV was estimated using the standard deviation of the mean R-R values and short-term HRV was estimated in five-minute periods using the SDNN index, a mean of five-minute standard deviations of the R-R intervals.ECG data was summarised as a mean for an individual patient across the study period. Hourly values were corrected to a 24-hour mean value where relevant. Where applicable, cut-off values of < 100 ms for SDNN and < 25 ms for triangular index (TI) were used.

A routine echocardiogram was performed following a previously validated protocol [27]. Standardized examinations included 2-D guided M-mode echo-cardiograms and selected 2-D recordings. Studies were performed using a high-quality commercially available echocardiograph (Sono 2000) equipped with Super-VHS video recorder. Measurements were made blindly by experienced physician readers. LV hypertrophy was considered to be present when LV mass index exceeded 116 g/m^2 ^for men and 104 g/m2 for women, and increased RWT was present when this ratio exceeded 0.43. Pulsed Doppler recordings of the transmitral flow velocities were obtained between the tips of the mitral valves, measuring early and atrial LV filling peak flow velocity as well as deceleration time of early diastolic transmitral flow. LV filling was considered normal when isovolumetric relaxation time was < 100 ms, deceleration time between 150 and 250 ms and ratio between early and atrial LV filling peak flow velocity between 1.0 and 1.5.

All subjects underwent a thorough medical examination to exclude any chronic illness i.e. diabetes, kidney disease, and other cardiovascular disorders. Consequently, blood samples were collected to measure glucose, urea and electrolytes, cholesterol and high-density lipoprotein (HDL)-cholesterol.

### Data analysis

We conducted statistical analysis to compare the differences in HRV, ABPM and cognitive function among the three groups. All results were expressed as mean value ± standard error of the mean (SEM). Post hoc test was used to compare differences between the three groups. To determine the relationship between HRV and other measured variables, univariate analysis followed with multiple regression analysis in a step-wise approach if variables demonstrated a significant relationship with HRV. Multiple regression analysis was used to assess association between HRV parameters and measures of clinic BP and ABPM. A p value of < 0.05 was considered to be significant. Correlation analysis between 24-hour RMSSD and other variables were performed to see if any relationship was apparent between groups.

## Results

The demographic and anthropometric characteristics of the participating subjects are shown in **Table **1. The mean age of the healthy control (mean ± SD; 31 ± 11 years) subjects was slightly less than that of the WCHT (42 ± 10 years) and BLH (40 ± 9 years) subjects. All three groups had normal LV mass indices (g/m^2^); control group (104 ± 9), WCHT (109 ± 5), and BLH subjects (mean ± SD; 110 ± 4). Similarly the three groups had normal diastolic function. No significant differences were observed in smoking habits (p = 0.39), alcohol consumption (p = 0.91) or family history of heart disease (p = 0.86) between the three groups.

**Table 1:** Demographic and Anthropometric Characteristics of the Subjects Stratified by Blood Pressure

Parameters	Normal BP (n = 20)	WCH (n = 19)	Borderline HTN (n = 30)	Total Number (*n *= 69)
	Mean ± SD	Mean ± SD	Mean ± SD	Mean ± SD

Age (years)	31 ± 11	42 ± 10	40 ± 9	38.2 ± 10.8

BMI (kg/m^2^)	23 ± 4	26 ± 6	28 ± 5	25.6 ± 5

Gender - (%)				

Male	34.3	40.0	25.7	50.7

Female	23.5	14.7	61.8	49.3

Lifestyle - (%)				

Smoking	25.0	30.0	45.0	29.0

Alcohol	27.8	27.8	44.4	78.3

FH of HD	29.7	29.7	40.6	53.6

BP; blood pressure, WCH; white coat hypertension, HTN; hypertension, BMI; body mass index, FH of HD; family history of heart disease.

The mean day-time ABPM (systolic and diastolic) values were significantly (p < 0.05) higher among WCHT, and more so among BLH as compared to the normal control subjects. When compared BP levels within each group, there was a significant fall in ABPM (day-time and night-time) compared to clinic BP levels especially among WCHT subjects. No significant differences were observed in both the clinic BP and the night-time ABPM measurements between the three groups as indicated in **Table **2.

**Table 2:** Clinic and ambulatory blood pressure monitoring (ABPM)

Parameters	Normal BP (N = 20)	WCH (N = 19)	Borderline HTN (N = 30)	Post-hoc analysis
	Mean ± SD	Mean ± SD	Mean ± SD	*p *value

Clinic SBP (mm Hg)	125 ± 18	161 ± 16	155 ± 16	NS

ABPM (day-time) SBP (mm Hg)	121 ± 13	131 ± 8	139 ± 6	< 0.05

ABPM (night-time) SBP (mm Hg)	111 ± 12	118 ± 11	121 ± 9	NS

Clinic DBP (mm Hg)	71 ± 8	93 ± 10	93 ± 11	NS

ABPM (day-time) DBP (mm Hg)	75 ± 7	82 ± 6	90 ± 6	< 0.05

ABPM (night-time) DBP (mm Hg)	65 ± 8	69 ± 10	77 ± 7	NS

Pulse	71 ± 9	86 ± 15	80 ± 19	< 0.05

BP: blood pressure, WCH: white coat hypertension, HTN: hypertension, SD; standard deviation.

### CANTAB computer and cognitive tests

A statistically significant difference was observed in the CANTAB computer test between the three groups. The mean scores obtained in the computer tests, the Alice Heim 4 (AH4), the PASAT and the State Anxiety Inventory (SAI) are summarized in **Table **3. Both WCHT and BLH subjects scored significantly higher than the control group in the PALT challenge score, and significantly lower than the control in the mean percentage of correct answers of the spatial recognition and memory task (SRMT). WCHT and more so BLH subjects had significantly lower RT than the control group. In the SSPT test WCHT were able to recall a longer sequence, on average, better than BLH subjects, but both groups performed worse than the control group. The same pattern was evident in the spatial working memory task (SWMT), where WCHT and BHL subjects achieved a significantly higher error than the control group. In summary, it was evident that subjects with WCHT performed poorly in memory tests (p = 0.001); whereas BLH subjects seemed to have slower reaction times (p = 0.001). While, no statistical relevance could be seen in the results of the AH4 verbal and numerical test or the PASAT, the mean scores in both tests for subjects with WCHTs showed the lowest scores. In the state anxiety inventory (SAI), both subjects with WCHT and BLH had significantly higher anxiety levels than the control group (p = 0.006). Multiple comparison between the three groups revealed significant differences between control subject scores in memory tasks in comparison with the other two groups (p = 0.004).

**Table 3:** Mean values of the Cambridge Neuropsychological Test Automated Battery (9CANTAB) computer tests and paper format tests

	Normal BP (N = 20)	WCH (N = 19)	Borderline HTN (N =30)	Post-hoc *p *value
**CANTAB computer tests**				

PALT ± SD	5 ± 5	15 ± 8	12 ± 8	0.069

SRMT ± SD	89 ± 6	71 ± 12	80 ±10	0.004

RT 11 ± SD	335 ± 54	393 ± 50	401 ±70	0.003

SSPT ± SD	7 ± 1	4 ± 2	3 ±3	0.060

SWMT ± SD	19 ± 12	36 ± 16	33 ±18	0.030

**Paper format tests**				

AH 4 ± SD	37 ± 13	30 ± 14	37 ±8	0.004

PASAT ± SD	44 ± 9	37 ± 13	42 ±11	0.003

SAI ± SD	29 ± 3	38 ± 10	36 ±8	0.006

BP; blood pressure, WCH; white coat hypertension, HTN; hypertension, CANTAB9; Nine Cambridge Neuropsychological Test Automated Battery (CANTAB) computer tests (9), PALT; paired associates learning task, SRMT; spatial recognition and memory task, RT11; reaction time, SSPT; spatial span test, SWMT; spatial working memory, AH4; Alice Heim 4, PASAT; paced auditory serial addition test, SAI; state anxiety inventory.

### Heart rate variability (HRV) - 24 hour RMSSD

We found a significant (p < 0.5) difference in the 24-hour RMSSD and SDNN, but not other HRV measures, between the three groups as shown in Table 4. Further, the 24-hour RMSSD was negatively correlated with clinic systolic readings i.e. high BP was associated with low HRV value (p = 0.01). Similar observations was made in the correlation between both 24-hour RMSSD and mean night-time diastolic BP and clinic diastolic BP (p = 0.001). No significant correlation was evident between 24-hour RMSSD and mean day-time systolic BP (p = 0.37), or mean day-time diastolic BP (p = 0.28) or mean night-time systolic BP (p = 0.06). Significant correlation was evident between 24-hour RMSSD and clinic pulse readings i.e. as pulse count per minute increases, HRV was reduced (p= 0.001). A very strong correlation was seen when 24-hour RMSSD was compared against 24- hour mean heart rate (MHR; p = 0.00).

**Table 4:** Comparison of heart rate variability measures between three groups; healthy normal subjects, white coat hypertension, and borderline hypertension subjects

HRV 24-hours	Normal BP (N = 20)	WCH (N = 19)	BLH (N = 30)	Post-hoc
	Mean ± SD	Mean ± SD	Mean ± SD	***p *value**

24hr MHR	77 ± 8	71 ± 10	77 ± 8	> 0.05

24hr Triangular Index	43 ± 10	39 ± 16	37 ± 11	> 0.05

SDNN	154 ± 50	149 ± 46	130 ± 33	< 0.05

24hr SDNN Index	71 ± 28	64 ± 25	58 ± 15	> 0.05

SDANN	130 ± 47	130 ± 41	113 ± 32	> 0.05

RMSSD	45 ± 28	33 ± 22	31 ± 14	< 0.05

24IT ±SD	43 ± 10	39 ± 16	37 ± 11	> 0.05

HRV; Heart rate variability, BP; blood pressure, WCH; White coat hypertension, BLH; borderline hypertension, MHR; Mean heart rate, SDNN; Standard deviation normal to normal interval, SDANN; Standard deviation average normal to normal interval, RMSSD; Square root of the mean squared differences of successive R-R intervals, 24IT; inspection time.

There was significant correlation between 24-hour RMSSD and computer CANTAB scores (in 4 out of 5). The paired associate learning task (PALT; p = 0.00) and spatial working memory test (SWMT; p = 0.04) revealed negative correlation with RMSSD i.e. as the scores increased, HRV decreased. However, although the spatial span test (SSPT) and the SRMT were significant (p = 0.02 and p = 0.03; respectively) the correlation was positive i.e. the test scores increased with increasing HRV. No significant correlation was found between 24-hour RMSSD and the state anxiety inventory (p = 0.27), or AH4 verbal and numerical test (p = 0.55) or the PASAT (p = 0.90).

Echocardiography revealed that compared to the normal control subjects, both WCHT and BLH subjects had higher LV mass indices and lower diastolic function parameters. All subjects were in good health and no chronic illness was reported as confirmed by routine laboratory analysis.

## Discussion

The primary aim of the project was to compare clinic BP, AMBP, cognition and HRV between healthy normal control subjects, WCHT, and BLH subjects.

Existing evidence shows that individuals with WCHT may develop persistent hypertension over a relatively short period of time [28]. These findings were based on the prognostic significance of ABPM in comparison with clinic BP measurement [29]. We report a significantly higher day-time ABPM (systolic and diastolic) values among WCHT, and more so among BLH subjects as compared to normal controls. The most important difference was seen in a sharp fall of BP (systolic and diastolic) between clinic-, day-time-, and night-time- BP readings among the WCHT, and though slightly less, among BLH subjects. Our results are consistent with available data in the literature [30], and may, in fact, correspond to the classical diagnostic findings of BP levels among WCHT subjects i.e. mean day-time BP of < 135/85 mm Hg, but > 140/90 mm Hg in the clinic according to the definition of the British Hypertension Society [24]. However, our data cannot confirm a foreseeable transition between WCHT to sustained hypertension. Generally, the BP observed between the groups served as a means of categorizing participants on the severity of hypertension thereby enabling comparisons to be made. The comparison of clinic and 24-hour BP has proved that WCHT was apparent in this sample. It also suggests that borderline subjects have higher sustained mean BPs over 24-hour. However, our study did not address, and therefore cannot explain longitudinal effects of these measurements. Hence, whether other factors could identify the future prognosis of these variables in patients with hypertension may rely on the evidence found when cognitive function and HRV were assessed.

Five out of the nine CANTAB computer tests revealed significant differences in performance between the three groups (post-hoc). It was evident from the mean scores that WCHT poorly performed in memory tests, whereas borderline subjects had slower reaction time, but both groups performed significantly worse than normotensives. These results were consistent with the observations of Elias and colleagues [31]. In contrast, other studies [32], have shown no relationship between BP and cognitive performance. These conflicting results may reflect several methodological differences in these studies. It was thought that the negative findings in the Farmer's study [32] was most likely due to BP measurements taken simultaneously with neuropsychological testing, or probably too few measurements were taken. Our findings agree with Elias *et al *of BP being inversely related to cognitive function. Despite the relatively small sample size, our results indicated a significant lack of performance in cognitive tests by subjects with WCHT and borderline when compared to controls.

The state anxiety inventory revealed that subjects with WCHT and BLH had significantly higher anxiety levels than normotensives. This may well account for this group's lack of performance in most of the cognitive tests and could clinically be the cause of the high BPs measured at the hospital i.e. WCHT. Psychological factors may be closely related to the development of hypertension [33]. However, all BP measurements and CANTAB computer test results remained significant even when data was stratified for sex, age and anxiety scores. Thus, BPs obtained and the computer test scores were independent of differences in age, sex and anxiety levels between the three groups. It can be speculated that these intermediate groups (WCHT and borderline subjects) will develop sustained hypertension associated with poor cognitive performance and high levels of anxiety. Such findings raise the possibility that controlling anxiety levels with these variables may reduce stress and consequently lower BP. Previous work [34] has suggested that relaxation training can increase parasympathetic tone on the heart thus reducing BP and increasing HRV. The results show that across neuropsychological testing, subjects with WCHT performed poorly in memory tests, whereas subjects with BLH showed slower reaction time, compared with controls.

Recently HRV has also been utilised as a predictor of hypertension [16]. The latter study indicated that control male participants with lower HRV had a greater risk of developing hypertension. Our study showed that subjects with borderline hypertension had lower HRV indices than subjects with WCHTs and normotensives (p > 0.05). It is worth noting that when 24-hour RMSSD was compared against sex, there was significant difference between the three groups (p < 0.05). This may indicate a greater effect of gender on the RMSSD values of subjects than the group type i.e. subject's BP. Therefore it can be predicted that if groups were gender matched, then significant and clinical differences in HRV domains may become apparent. The 24-hour RMSSD correlated significantly with clinic systolic, clinic diastolic, mean night-time diastolic, clinic pulse and mean heart rate values. All these correlations were negative. Thus an increased BP and pulse values was associated with a decreased 24-hour RMSSD (and hence HRV) and; a finding that supports the possible association between high BP and low HRV.

Significant correlation was also evident between four out of the five computer cognition tests. Negative correlation between 24-hour RMSSD, PALT and the SWMT was evident. This suggests that greater number of errors in these tests (i.e. poor cognition) may be linked to low HRV. However, there was positive correlation between 24-hour RMSSD and SSPT and the SRMT. The scores for these tests were based on the number of correct answers, so the positive correlation suggests that low scores (i.e. poor cognition) are linked to low HRV. This correlation entails significant association of low HRV with high BP and poor cognitive performance in this sample, but the differences between the groups were not significant.

Echo cardiography revealed that compared to control subjects, both WCHT and BLH subjects had normal but thicker left ventricular (LV) mass and normal but reduced diastolic function. This is important because detecting signs of organ damage in WCHT may further help physicians to correctly plan antihypertensive therapy, particularly, among patients at higher risk of CVD and other organ damage i.e. CKD from excessive BP reduction [35].

Taken together, the findings from this study cannot answer the question of whether to treat or not treat WCHT, in part, because of the cross-sectional nature of the study, and indeed the relatively smaller sample size. Nonetheless, the current study may provide new insights on the basis of associated new surrogate risks i.e. cognitive and HRV dysfunction

### Study limitations

Firstly, we did not record the exact time of BP measurements in the clinic. Moreover, no standardized measures were taken to reduce the stressor "pressor effects" of the clinic setting. Secondly, unmeasured confounders such as education [36], may have affected the current cognitive function results. Longitudinal data may be required to produce a clearer understanding of the effects of cognition on clinic and ambulatory BP values over time. Thirdly, the small sample size used in our study may have concealed the true differences in HRV among the groups. The lack of any relationship between group type and HRV was most likely due to the lack of group participants and failure of gender-matching.

## Conclusions

WCHT subjects performed poorly in memory tests, whereas BLH subjects had slower reaction time, but both groups performed significantly worse than controls. Autonomic functions reflected by HRV indices were lower in WCHT and BLH subjects in contrast to controls though not statistically significant. We noted that the structural changes found in the left ventricle among the WCHT subjects; including increased normal LV mass, and decreased normal diastolic function may contribute to the overall risk for cardiovascular disease and mortality. Therefore, WCHT may not be considered a benign condition, but rather a condition that can result in LV damage. Longitudinal studies of patients with WCHT should clarify the transient, persistent or the progressive nature of this condition.

## Competing interests

The authors declare that they have no competing interests.

## Authors' contributions

AS Developed the research concept and design, collected the data, performed data analysis and interpretation, conducted literature review on the topic, drafted the initial manuscript, and provided subsequent critical review and approval of the manuscript. AA Substantially contributed to the data analysis and interpretation, co-authored and critically reviewed the manuscript.

## Pre-publication history

The pre-publication history for this paper can be accessed here:

http://www.biomedcentral.com/1471-2261/11/3/prepub
